# Supplementary material for: CHARMM-GUI Multicomponent Assembler for modeling and simulation of complex multicomponent systems
Source: Nat Commun. 2024 Jun 27;15:5459. doi: 10.1038/s41467-024-49700-4 (PMC11211406; doi:10.1038/s41467-024-49700-4)
Supplement: Supplementary file 1 — Supplementary Information [file 41467_2024_49700_MOESM1_ESM.pdf]

Supplementary Information

CHARMM-GUI Multicomponent Assembler for Modeling and Simulation of Complex Multicomponent Systems

Authors

Nathan R. Kern[1], Jumin Lee[2], Yeol Kyo Choi[2], and Wonpil Im[1,2,3*]

[1]Department of Computer Science & Engineering, Lehigh University, Bethlehem, PA, USA
[2]Department of Biological Sciences, Lehigh University, Bethlehem, PA, USA
[3]Department of Bioengineering, Lehigh University, Bethlehem, PA, USA.

*Corresponding author: wonpil@lehigh.edu

Supplementary Methods

CO_2 Diffusion

We used the methods in Im and Roux's 2002 study[1] to calculate position dependent diffusion coefficients along the Z axis (D(z)) for embedded CO_2 with lag times (τ) from 1–10 ns (**Figure S6**). Mean and standard errors were calculated from the same bin positions across different simulation replicas, and diffusion in the polymer center (**Table S1**) was calculated by averaging all observations within bins located between -15 Å < z < 15 Å.

We found that D(z) varies substantially by Z position, even within the polymer center, as CO_2 molecules find defects in the polymer structure where local diffusion is increased on short time scales. The mean of diffusion across bins in the polymer center was calculated to be almost 2x larger for PET_{95} (7.8 ± 2.2 $cm^2/s \times 10^{-8}$) than PEF_{95} (4.0 ± 0.3 $cm^2/s \times 10^{-8}$), possibly due to there being more defects within the PET_{95} structure. Of the studies of CO_2 diffusion in PET we surveyed (**Table S1**), the most similar rate is in a simulation (2.43 $cm^2/s \times 10^{-7}$ at 25 °C) which used lag times on the order of picoseconds. An experimental study[2] reported much slower CO_2 diffusion (7.2 ± 2 $cm^2/s \times 10^{-11}$ and 2.2 ± 0.4 $cm^2/s \times 10^{-9}$ for PEF and PET, respectively), however their finding that CO_2 diffuses more slowly through PEF than PET is consistent with ours. That study measured permeation more directly via changes in atmospheric pressure across a macroscopic plastic barrier, whereas our study measures diffusion by tracking individual particles in a microscopic barrier.

To compare with other diffusion studies, it should be noted that the scale of D(z) depends on τ; the smaller timescales of simulations necessitate smaller τ values, which tends to overestimate long-term diffusion compared to experiments. The utility of simulation studies in this domain is thus by comparison of relative diffusion rates, rather than calculation of absolute diffusion rates.

Mica + POPC Preparation

In Nanomaterial Modeler (https://charmm-gui.org/input/nanomaterial), we selected Mica > Muscovite from the Nanomaterial Type menu. In Box Options, we entered the approximate lengths of X = 100, Y = 100, and Z = 30. Nanomaterial Modeler automatically rounds up to the nearest unit cell size for the selected material (displayed in parentheses), which in this case is X = 103.8 Å, Y = 108.2 Å, and Z = 30.1 Å.

After clicking "Next", it took about 2 minutes to build this mica model. On the next page, we ignored all options and selected "download .tgz" to obtain the mica-only model. We extracted the `.tgz` archive, located the files named `step1_nanomaterial.psf` and `step1_nanomaterial.crd`, and renamed them to `muscovite.psf` and `muscovite.crd`, respectively. We also used the values for A, B, and C in `step1_nanomaterial.str` as the reference values for Multicomponent Assembler.

In Multicomponent Assembler, we uploaded the muscovite PSF/CRD files, and clicked "Next". On the size determination page (STEP 1), we set the component type of muscovite to "Periodic". In the Periodic Component Size table, we used the values A and B saved in `step1_nanomaterial.str` for the length of X and Y, respectively (i.e., X = 103.836, Y = 108.1836) and estimated Z as ZMAX - ZMIN from the same file (i.e., Z = 29.92). We estimated the thickness of a pure POPC membrane as 46.86 and set the water thickness to 23.43. In the Periodic Components table, we clicked "Set Position" to leave space for a 10 Å thick layer of water between the bottom leaflet of POPC and muscovite by using the default constraint type and positioning type ("Fixed Z position" and "Center of mass", respectively) and set the component position to 48.33. After clicking "Calculate System Size" and "Next", we set the ratio of POPC to 1 in both leaflets, clicked "Show the system info", and clicked "Next". On the next page, we set the ion placing method to "Monte-Carlo" and used 0.15 M KCl and clicked "Next". On the Solvent Options page, we used default values, clicked "Calculate Solvent Composition", then clicked "Next". On the Input Options page, we selected "OpenMM" input generation, "NPT ensemble", and a temperature of 298.15 K. After input generation completed, we downloaded the result with "download .tgz".

We followed the procedure in the previous paragraph to create the Mica + POPC models with a 20 Å and 30 Å water gap, except that the component position of muscovite was set to 58.33 and 68.33, respectively. Since we did not use the default equilibration scheme, we made the following changes to each model's OpenMM input options. In `step6.3_equilibration.inp` through `step6.6_equilibration.inp`, we changed "pcouple" from "no" to "yes" and added the following lines:

```
p_ref   = 1.0, 1.0, 1.0  # Pressure (Pref or Pxx, Pyy, Pzz; bar)
p_type  = anisotropic    # MonteCarloBarostat type
p_scale = XYZ
p_freq  = 100
```

Additionally, for `step6.6_equilibration.inp`, we increased the number of steps (nstep) from "250000" to "1250000".

Algorithm 1. **Packing optimization.** A greedy search of translation and rotation space for a given component (comp). cmax is the maximum allowed number of collisions. dcut is the distance around any atom in comp to check for collisions. $\delta\theta$ is the rotation increment. θ_0 is the initial rotation angle along each axis. θ_{max} is the maximum rotation angle along each axis. Collision-detection ignores CG particles if any have not yet been replaced.

```
GreedyTransRotSearch(comp, cmax, dcut, δxyz, δθ, θ0, θmax):
    let best = (Infinity)
    if component type is membrane
        for all (tx, ty) in 2-fold Cartesian product of {-δxyz, 0, δxyz}
            translate comp by tx, ty
            if DoRotation(comp, cmax, dcut, δθ, θ0 , θmax, tx, ty, 0, best)
                return
            endif
            undo translation
        endfor
    else
        for all (tx, ty, tz) in 3-fold Cartesian product of {-δxyz, 0, δxyz}
            translate comp by tx, ty, tz
            if DoRotation(comp, cmax, dcut, δθ, θ0, θmax, tx, ty, tz, best)
                return
            endif
            undo translation
        endfor
    endif
    translate comp by best[1], best[2], best[3] // if this line reached, no
    rotate comp by best[4], best[5], best[6]    // trans/rot is ≤ cmax;
endfunc                                         // restore best trans/rot
DoRotation(comp, cmax, dcut, δθ, θ0, θmax, tx, ty, tz, best):
    let angles = {}
    for i=0 to floor((θmax - θ0) / δθ)
        append (θ0 + i*δθ) to angles
    endfor
    if component type is membrane
        angles = {(0, 0, θz) forall θz in angles}
    else
        angles = 3-fold Cartesian product of angles
    endif
    forall (θx, θy, θz) in angles
        rotate comp to (θx, θy, θz) along (x, y, z) axes
        let collisions = number of atoms of comp within dcut of
                  atoms not in cmax, including image atoms
        if component type is not membrane
            collisions += number of atoms of comp within dcut of
                   membrane exclusion region
        endif
        if collisions < best[0]
            best = (collisions, tx, ty, tz, θx, θy, θz)
        endif
        if collisions ≤ cmax then return true
    endfor
    undo rotation
    return false
endfunc
```

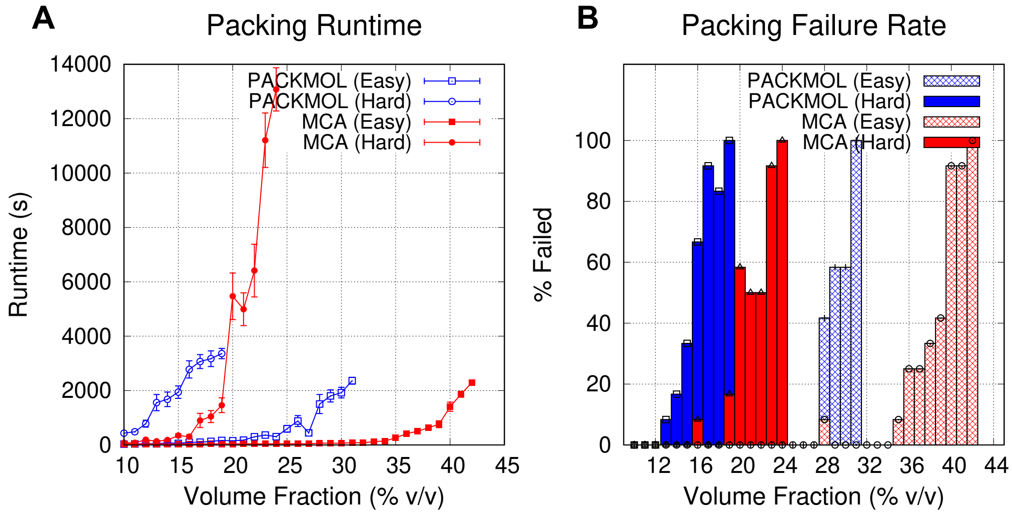

Supplementary Figure 1. **Comparison of performance between PACKMOL and MCA.** In both figures, n = 12 packing attempts. (A) Mean ± SEM runtime of packing tasks at a given volume fraction (% v/v). The last point in each line is the v/v at which all packing attempts failed. Unfilled blue square: PACKMOL (Easy); unfilled blue circle: PACKMOL (Hard); filled red square: MCA (Easy); filled red circle: MCA (Hard). (B) The fraction of packing attempts that fail. Only the lowest v/v resulting in 100% failure is shown for each combination of program and molecule set. Cross: PACKMOL (Easy); square: PACKMOL (Hard); circle: MCA (Easy); triangle: MCA (Hard).

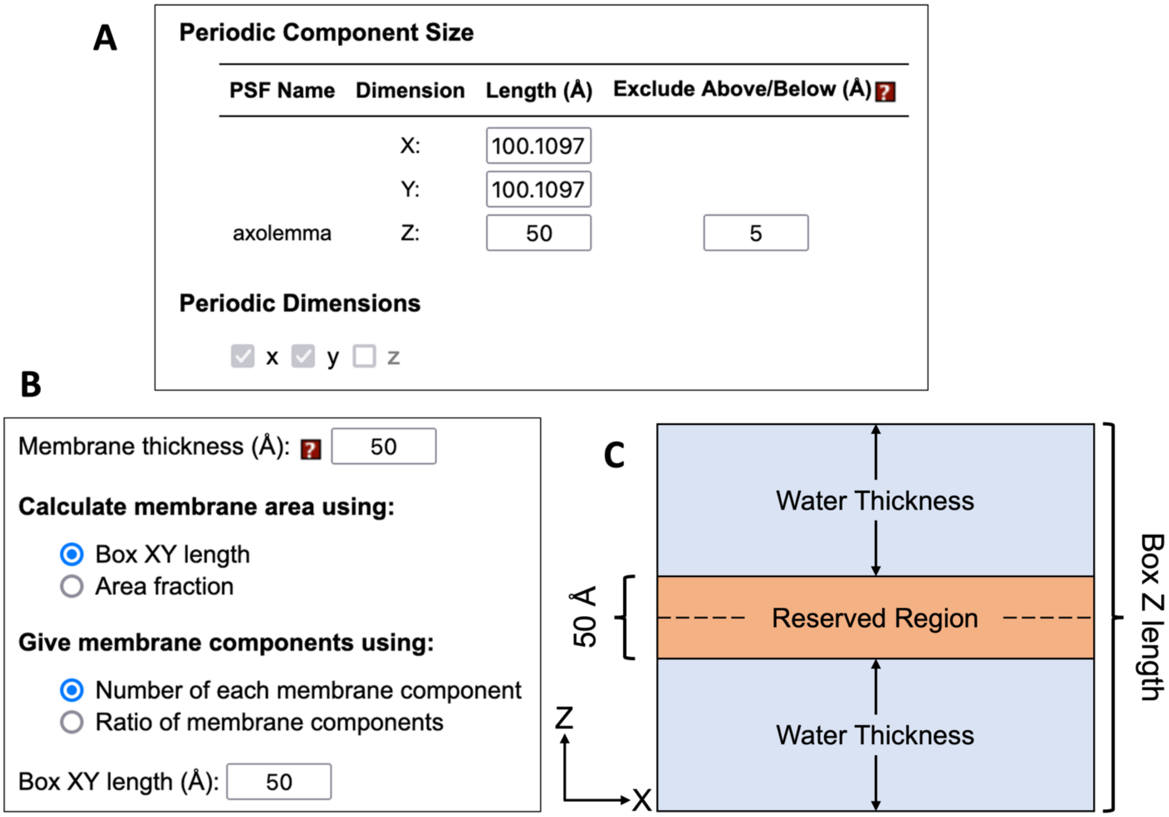

Supplementary Figure 2. **Size determination options for systems with a membrane or periodic component.** (A) The presence of a periodic component shows the periodic component size table, in which the user provides exact X and Y dimensions and an approximate Z dimension. If present, multiple periodic components must share X and Y dimensions. (B) The user can provide the exact length of the X dimension (X=Y) and estimate the membrane thickness along the Z axis. "Membrane thickness" is functionally equivalent to the periodic component Z length. (C) The X, Y, and Z lengths of any or periodic component membrane are used to reserve a box-shaped region for that component. Other components are initialized outside reserved regions and cannot enter them during packing.

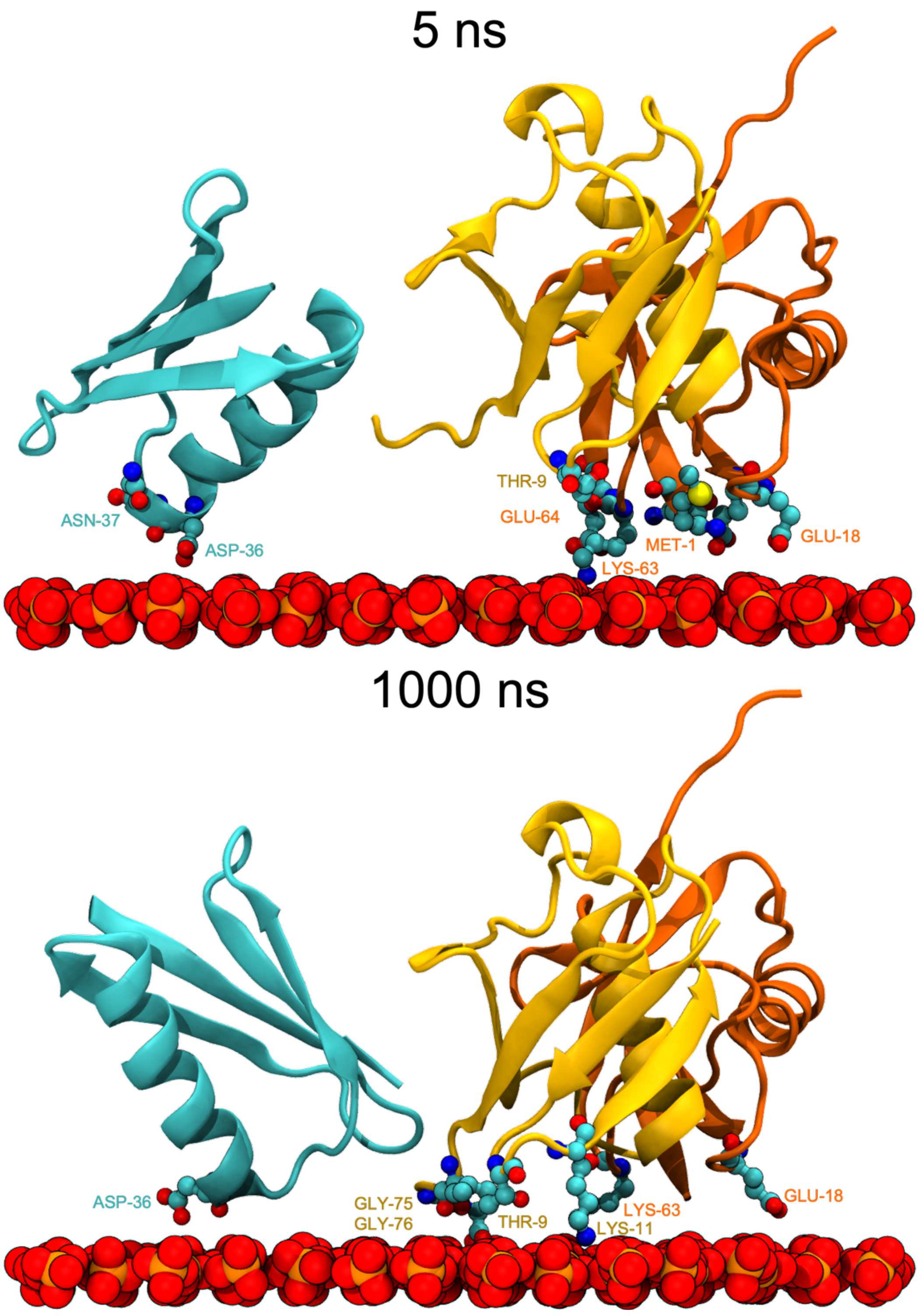

Supplementary Figure 3. **Example protein cluster with long-lived contact to HAP.** The contact event shown begins at 5 ns and lasts for the rest of the 1 μs simulation. The cluster consists of one protein G (PDB: 3GB1, cyan) and two ubiquitin (PDB: 1UBQ, yellow and orange). Proteins in this system occupied 10% of solvent volume. These representative structures were identified visually.

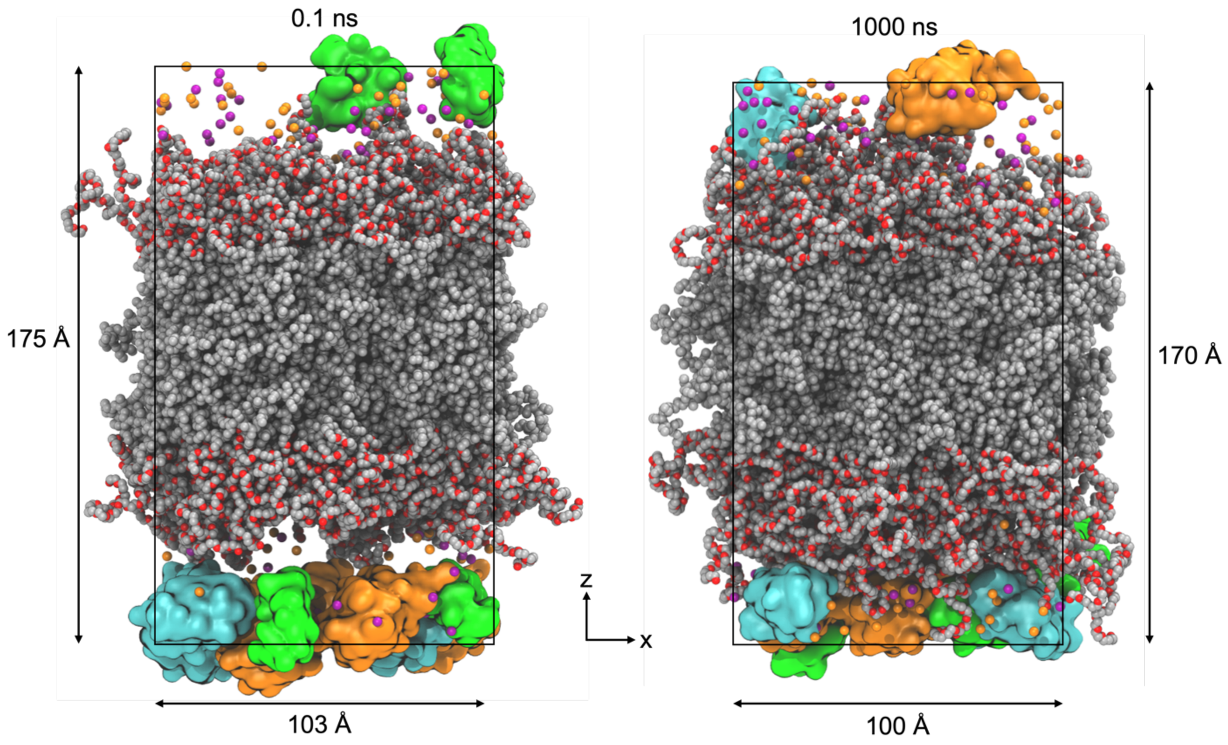

Supplementary Figure 4. **Initial and final snapshots of $EO_{40}EE_{37}$ polymer membrane with proteins.** The color scheme is the same as in Figure 1d. $EO_{40}EE_{37}$: polyethylene oxide-poly(ethylethylene).

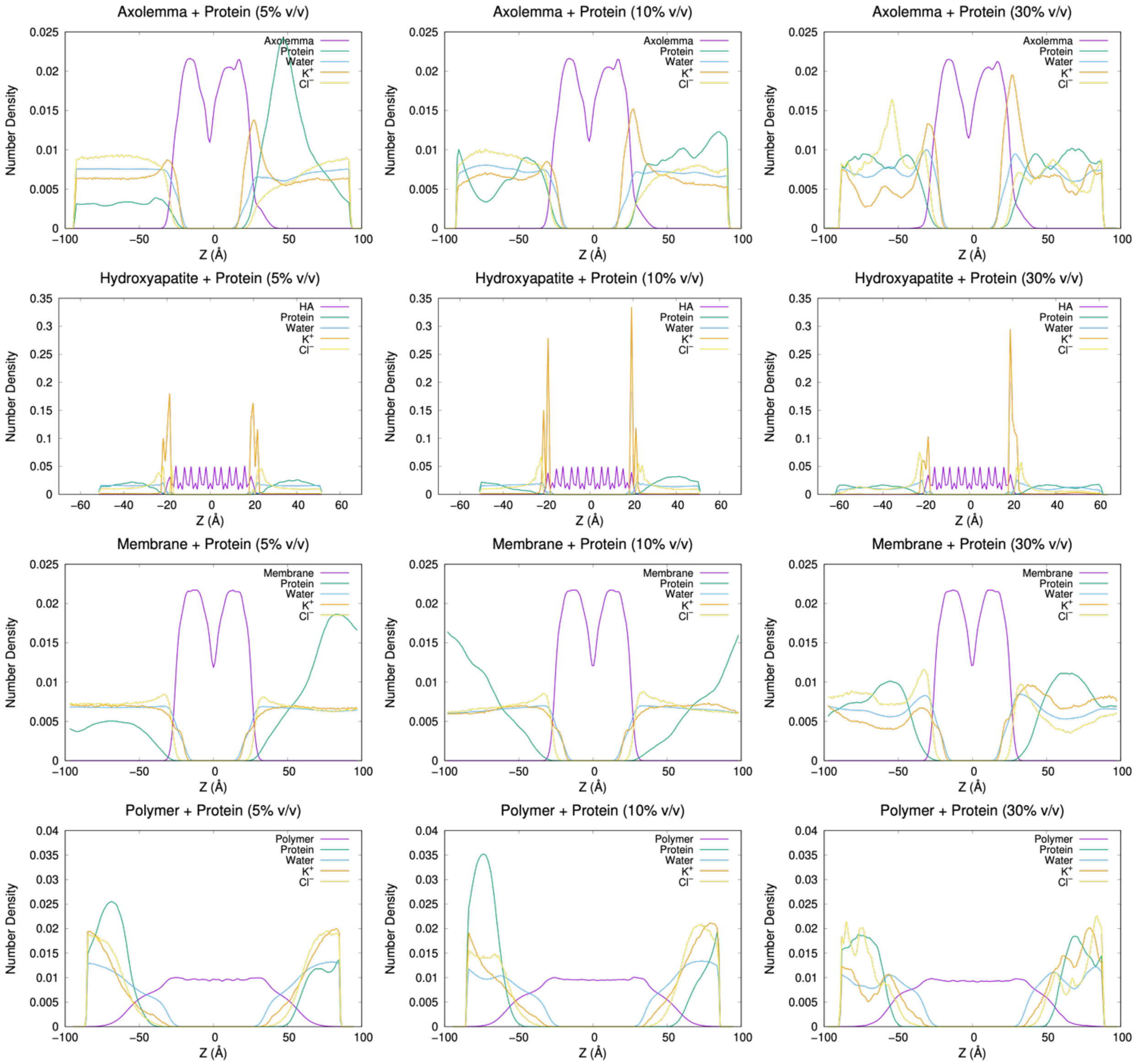

Supplementary Figure 5. **Z density profiles of all systems containing a membrane-like component and proteins.** Values are averaged across the whole simulation and plotted with a bin size of 1 Å. Bin heights are normalized to sum to 1. HA: hydroxyapatite.

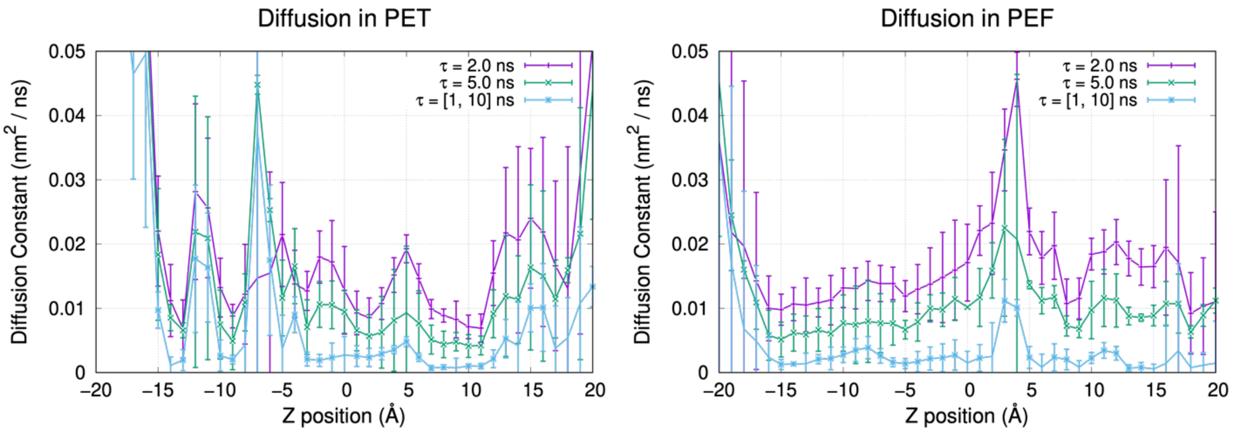

Supplementary Figure 6. **Position dependent diffusion profiles of CO_2 through PET_{95} and PEF_{95} in its approximate center.** The purple and green lines were calculated from the equation $D = \mathrm{MSD}(\tau) / (2\tau)$ with lag times of 2 and 5 ns, where D is diffusion, $\mathrm{MSD}(\tau)$ is the measured mean square displacement of CO_2 when using a given lag time τ. The blue lines result from linear regression of the same equation with all lag times (at 0.1 ns intervals) from 1.0 to 10.0 ns. Error bars represent the standard error of the mean across all simulation replicas within each bin. PET: polyethylene terephthalate. PEF: polyethylene 2,5-furandicarboxylate.

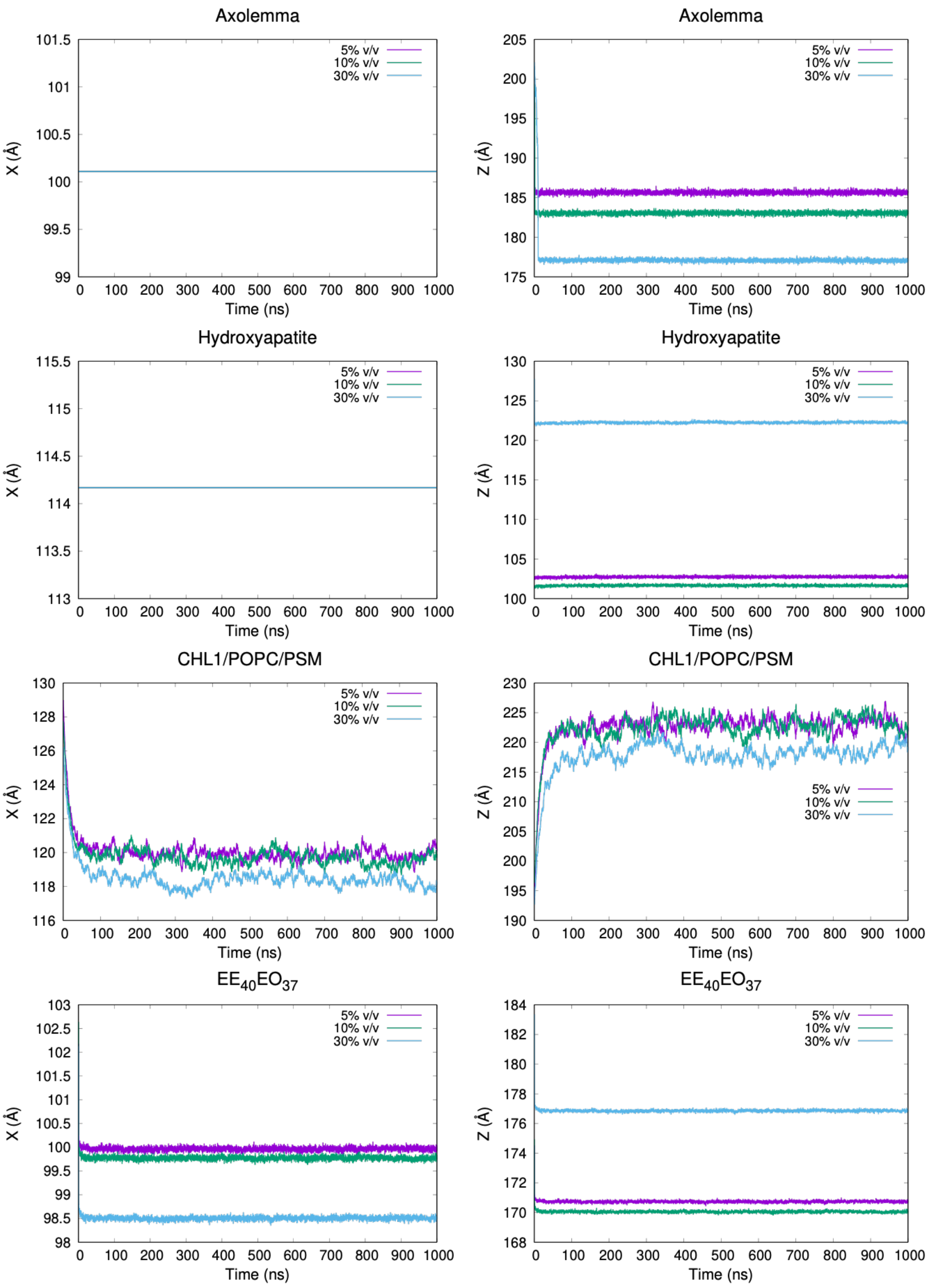

Supplementary Figure 7. **System dimensions of all systems containing a membrane-like component and proteins during production runs.** Y dimensions are omitted because X = Y. CHL1: cholesterol, POPC: 1-palmitoyl-2-oleoyl-phosphatidylcholine, PSM: palmitoylsphingomyelin (PSM). $EO_{40}EE_{37}$: polyethylene oxide-poly(ethylethylene).

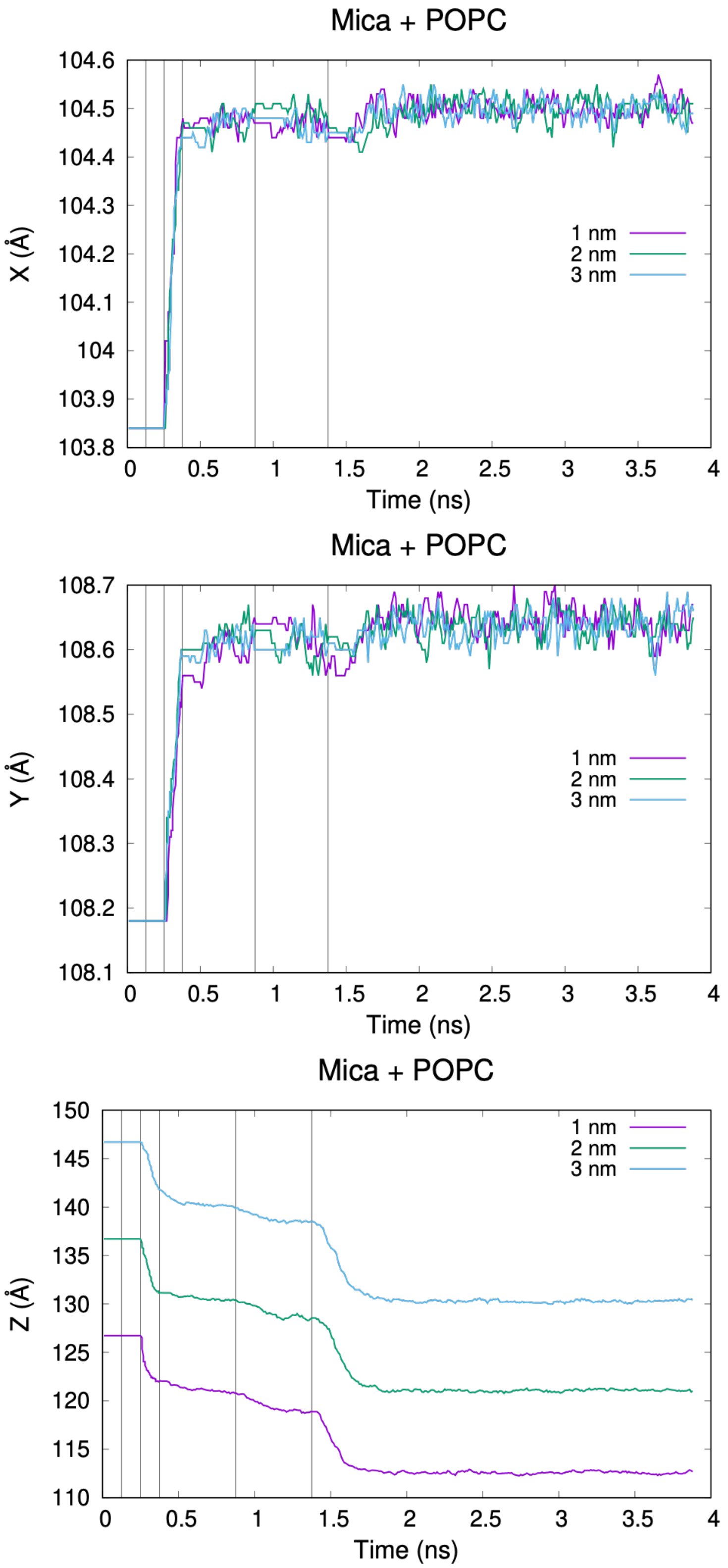

Supplementary Figure 8. **System dimensions of mica + POPC systems during equilibration.** Vertical bars indicate the simulation times at which the strength of restraints was decreased. POPC: 1-palmitoyl-2-oleoyl-phosphatidylcholine.

Supplementary Table 1. **Selected diffusion constants in this and other studies.** Error/confidence intervals are shown where included in the original study.

Reference	Study Type	Polymer	D_{CO2} (cm^2/s)	Temperature
[2]	Experiment	PEF	$(7.2 \pm 2) \times 10^{-11}$	35 °C
[2]	Experiment	PET	$(2.2 \pm 0.4) \times 10^{-9}$	35 °C
[3]	Experiment	PET	1×10^{-9}	STP
[4]	Simulation	PET	2.43×10^{-7}	25 °C
This study	Simulation	PEF	$(1.7 \pm 0.1) \times 10^{-7}$	25 °C
This study	Simulation	PET	$(1.5 \pm 0.1) \times 10^{-7}$	25 °C

Supplementary Table 2. **Analysis of various programs for multicomponent molecular assemblies.** “Special script” refers to a scripting language created specifically for a given modeling program. Topology preparation is “automatic” if individual molecule topologies can be inferred or read from a database and “manual” if they must be provided separately by the user.

	MCA	Pysimm	Moltemplate	PACKMOL	Polyply
User Interface	GUI or CHARMM script	Python API	Special script	Special script	Special script
Supported Simulation Programs	GROMACS CHARMM OpenMM Amber GENESIS Desmond	LAMMPS CASSANDRA	LAMMPS	N/A	GROMACS
Supported Force Fields	CHARMM AMBER	CHARMM AMBER GAFF DREIDING PCFF	OPLS COMPASS MARTINI GAFF DREIDING	N/A	MARTINI GROMOS AMBER OPLS
Topology Preparation	Automatic	Automatic	Manual	N/A	Manual

Supplementary Table 3. **Molecules used in benchmark.** 10 copies (easy) and 8 copies (hard) of each molecule were packed into cubic geometries.

Test Name	PDB ID	Asphericity	# Residues	Volume (Å^3)
Easy	1ubq	0.07	76	9.37×10^3
	1vii	0.14	36	4.45×10^3
	3gb1	0.15	56	6.62×10^3
Hard	1mjc	0.02	69	7.84×10^3
	3gb1	0.14	56	6.62×10^3
	1vii	0.15	36	4.45×10^3
	6y3g	0.41	87	2.15×10^4
	2hac	0.48	60	7.78×10^3

Supplementary Table 4. **System configurations used in this study.**

System Type	Protein v/v	Component	Copies	Initial System Dimensions	Production Runtime
Solvated Proteins	5%	1UBQ	5	$(153.32\ Å)^3$	1 μs
		1VII	5		
		3GB1	5		
	10%	1UBQ	10	$(154.06\ Å)^3$	1 μs
		1VII	10		
		3GB1	10		
	30%	1UBQ	10	$(106.31\ Å)^3$	1 μs
		1VII	10		
		3GB1	10		
Proteins + Membrane	5%	1UBQ	4	$(134.21\ Å)^2$ x 193.71 Å	1 μs
		1VII	4		
		3GB1	4		
		Cholesterol	110:111		
		POPC	110:111		
		PSM	110:111		
	10%	1UBQ	8	$(134.18\ Å)^2$ x 195.34 Å	1 μs
		1VII	8		
		3GB1	8		
		Cholesterol	110:110		
		POPC	110:110		
		PSM	110:110		
	30%	1UBQ	23	$(133.74\ Å)^2$ x 194.83 Å	1 μs
		1VII	23		
		3GB1	23		
		Cholesterol	108:107		
		POPC	108:107		
		PSM	108:107		
Proteins + Axolemma	5%	1UBQ	5	$(100.11\ Å)^2$ x 202.99 Å	1 μs
		1VII	5		
		3GB1	5		
		Axolemma	1		
	10%	1UBQ	10	$(100.11\ Å)^2$ x 202.99 Å	1 μs
		1VII	10		
		3GB1	10		
		Axolemma	1		
	30%	1UBQ	10	$(100.11\ Å)^2$ x 206.2 Å	1 μs
		1VII	10		
		3GB1	10		
		Axolemma	1		

System Type	Protein v/v	Component	Copies	Initial System Dimensions	Production Runtime
Proteins + HAP	5%	1UBQ<br>1VII<br>3GB1<br>HAP	2<br>2<br>2<br>1	103.59 x<br>114.17 x<br>111.06 $Å^3$	1 μs
	10%	1UBQ<br>1VII<br>3GB1<br>HAP	4<br>4<br>4<br>1	103.59 x<br>114.17 x<br>111.06 $Å^3$	1 μs
	30%	1UBQ<br>1VII<br>3GB1<br>HAP	17<br>17<br>17<br>1	103.59 x<br>114.17 x<br>111.06 $Å^3$	1 μs
Proteins + $EO_{40}EE_{37}$	5%	1UBQ<br>1VII<br>3GB1<br>$EO_{40}EE_{37}$	2<br>2<br>2<br>1	$(107.88\ Å)^2$ x<br>189.51 Å	1 μs
	10%	1UBQ<br>1VII<br>3GB1<br>$EO_{40}EE_{37}$	4<br>4<br>4<br>1	$(107.88\ Å)^2$ x<br>189.51 Å	1 μs
	30%	1UBQ<br>1VII<br>3GB1<br>$EO_{40}EE_{37}$	13<br>13<br>13<br>1	$(107.88\ Å)^2$ x<br>196.04 Å	1 μs

System Type*	CO_2 Density	Component	Copies	Initial System Dimensions	Production Runtime
PET_{95} + CO_2	1.98 g L^{-1}	PET_{95}<br>CO_2	1<br>64	$(108.4)^2$<br>x 300.00 Å	2 μs
PEF_{95} + CO_2	1.98 g L^{-1}	PEF_{95}<br>CO_2	1<br>64	$(108.4\ Å)^2$ x<br>300.00 Å	2 μs

*3 replicas of each PET and PEF system were built with the same parameters.

The TIP3P water model and 0.15 M KCl are used in all aqueous systems. All proteins and lipids are modeled with the CHARMM36(m) FF; polymers and CO_2 are modeled with CGenFF; mica and hydroxyapatite are modeled with the INTERFACE FF.

References

1. Im, W. & Roux, B. Ions and Counterions in a Biological Channel: A Molecular Dynamics Simulation of OmpF Porin from Escherichia coli in an Explicit Membrane with 1M KCl Aqueous Salt Solution. *J. Mol. Biol.* **319**, 1177–1197 (2002).
2. Burgess, S. K., Kriegel, R. M. & Koros, W. J. Carbon Dioxide Sorption and Transport in Amorphous Poly(ethylene furanoate). *Macromolecules* **48**, 2184–2193 (2015).
3. Okuji, S. *et al.* Surface modification of polymeric substrates by plasma-based ion implantation. *Nucl. Instrum. Methods Phys. Res. Sect. B Beam Interact. Mater. At.* **242**, 353–356 (2006).
4. Liao, L.-Q., Fu, Y.-Z., Liang, X.-Y., Mei, L.-Y. & Liu, Y.-Q. Diffusion of CO_2 Molecules in Polyethylene Terephthalate/Polylactide Blends Estimated by Molecular Dynamics Simulations. *Bull. Korean Chem. Soc.* **34**, 753–758 (2013).
